# Investigating the phenology and interactions of competitive plant species co-occurring with invasive *Lantana camara* in Indian Himalayan Region

**DOI:** 10.1038/s41598-023-50287-x

**Published:** 2024-01-03

**Authors:** Abhishek Kumar, Sanjay Singh, Dinesh Kumar, Ram Kumar Singh, Ajay Kumar Gupta, Kangujam Premkumar, Harish Bahadur Chand, Anil Kumar Kewat

**Affiliations:** 1 Forest Ecology and Climate Change Division, Forest Research Institute, Dehradun, India; 2 Centre of Excellence for Sustainable Land Management, Indian Council of Forestry Research and Education, Dehradun, India; 3 Silviculture and Forest Management Division, Forest Research Institute, Dehradun, India; 4 G.B Pant National Institute of Himalayan Environment, Ladakh Regional Centre, Leh, India

**Keywords:** Ecology, Plant sciences, Climate sciences, Environmental sciences

## Abstract

Invasive plant species are considered one of the significant drivers of habitat loss, leading to biodiversity loss. They have also been observed to alter the local ecology, resulting in a decline of native flora. The management of invasive species is widely recognised as one of the most severe challenges to biodiversity conservation. The International Union for Conservation of Nature (IUCN) considers *Lantana camara,* as one of the ten worst weeds. Over time, native and indigenous species may evolve to co-exist or compete with invasive species, reducing invader fitness. It is observed that species competition fluctuates throughout environmental gradients, life phases, and abundances. Hence, competition outcome is very context-dependent. To address this challenge, we conducted a comprehensive study in three phases: we identified native species coexisting with *Lantana* in their natural habitats in the Doon Valley (Phase I) and documented the phenotypic traits of selected coexisting species using the Landmark BBCH (Biologische Bun-desantalt, Bundessortenamt und Chemische Industrie) scale, revealing the phenological growth patterns of selected co-existing species (Phase II). This was followed by conducting pot (Phase IIIa) and field (Phase IIIb) experiments to study the interactions between them. Notably, *Justicia adhatoda*, *Broussonetia papyrifera*, *Pongamia pinnata*, *Urtica dioica* and *Bauhinia variegata* demonstrated promising results in both pot and field conditions. Furthermore, after the mechanical removal of *Lantana* and prior to the plantation in the field experiments, four native grass species were introduced using the seed ball method. Among these, *Pennisetum pedicellatum* and *Sorghum halpense* exhibited prompt regeneration and effectively colonised the field, densely covering the cleared area. The study provides a comprehensive management plan for the restoration of *Lantana* affected areas through competition using native species. This study utilizes phenological assessment for native plant selection using reclamation from native grasses and proposes a management plan for combating invasive *Lantana*.

## Introduction

**Figure 1:**
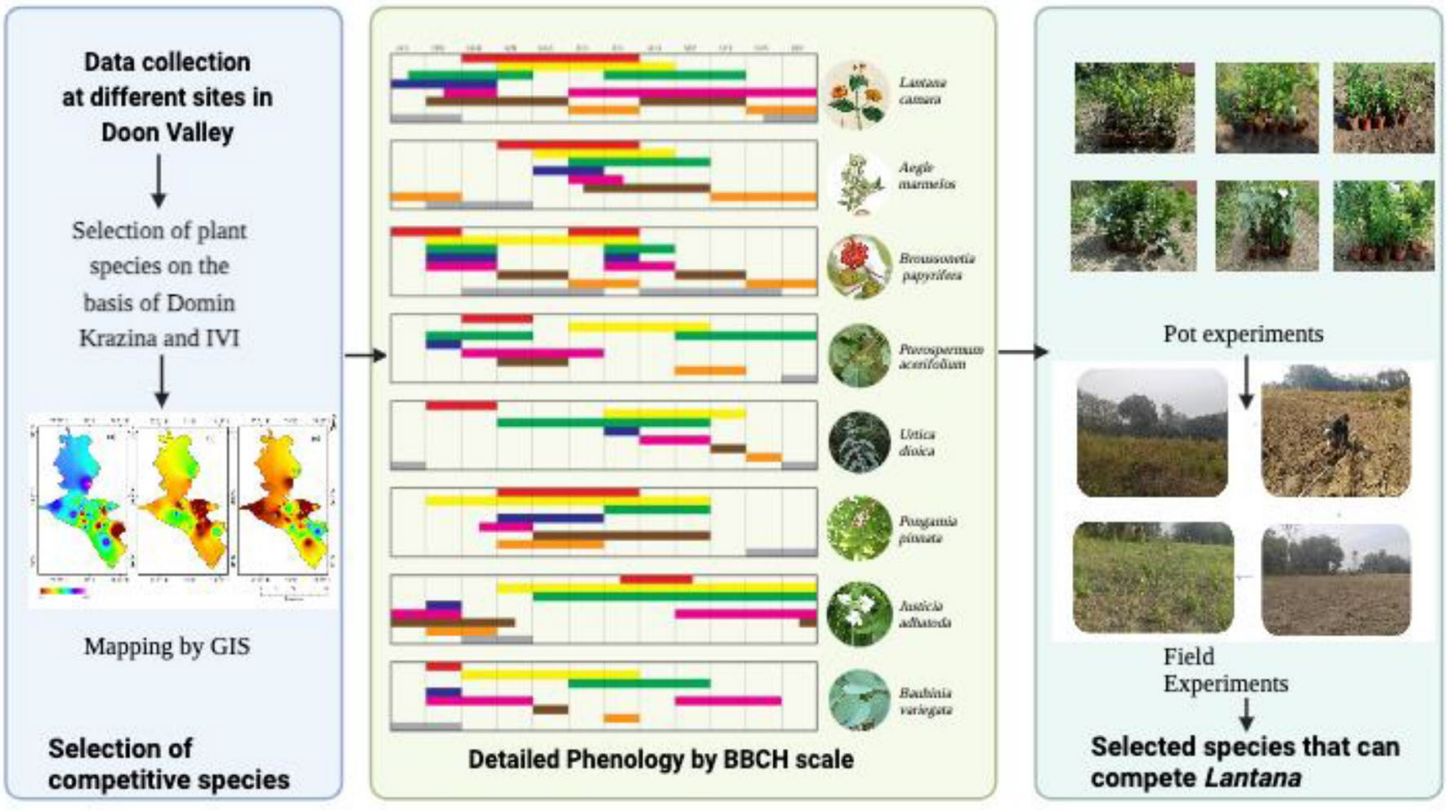
An overview of research**.** This figure depicts that study is divided into three phases: (i) selection of competitive species followed by (ii) studying the detailed phenology by BBCH scale and (iii) selection of native species (by studying phenology) that compete with *Lantana* by performing pot and field experiments.

Invasive Alien Species (IAS) are non-native species which have been introduced outside their native range either naturally or via anthropogenic means and pose serious impacts on biodiversity throughout the globe^1–3^. Several theories explain the mechanisms of plant invasions^4,5^. When invasive plant species (IAS) breach natural dispersal barriers, their natural enemies lose control of them, also known as the enemy release (Enemy release hypothesis)^6^. In the absence of natural enemies, IAS inhibits a high rate of propagation and establishment. It is a common notion that IAS outcompete the native competitors in their habitat, (Evolution of increased competitive ability hypothesis)^7^. Research has established that the high competitive ability of IAS has resulted in habitat fragmentation and loss of native biodiversity through competition^8,9^. IAS disrupts environmental equilibrium due to both direct and indirect drivers, reducing biodiversity^10^. IAS uses a variety of strategies to establish itself in new environments. The "new weapon hypothesis^10,11^, and "biotic resistance hypothesis"^12^ have speculated that allelochemicals emitted by a root or leaf of IAS may restrict the growth and establishment of native species. There are scientific evidence of IAS interactions where one invasive species may facilitate the invasion of another invasive, also known as “invasional meltdown”^13^. By modifying the bio-physical character of the host community and propagule pressure, invader–invader interactions could affect the success of future invaders^14^. However, all these theories have remained controversial. Some researchers reported that allelopathy can help native species withstand invasion by improving their biotic resistance against invasive^15^ (Fig. 1).

The limiting similarity hypothesis states that plants compete among themselves when they occupy similar niche, and co-exists when the niche differs. On the contrary dominant species enhance environmental stressors, and the nature of interactions between the plants can be modified by them^16^. This states that related species flourish well when they co-exist based on functional traits according to phylogeny. However, some researchers argued that trait-based niche descriptions could overlook interaction patterns between distinct species^17^.

Plant phenology is related to ecosystem processes and functions^18^. It is impacted by habitat and climate, which may determine the timing and frequency of phenological events in the life-history of the plant. As a result, phenological studies in many biomes has received much attention in recent years^19^. The phenology of an organism is an essential biological characteristic that determines its survival in the community^20^. It ultimately depends on the plant's inherent adaptability and its exposure to variations in the habitat conditions over the long run. Studying phenology finds its relevance as it describes the empirical discrete evidence^21,22^ that affects the topology of plant interactions. Studying the flowering patterns^23^ related to climatic factors has been of interest to ecologists to interpret the behavior of non-native species. Similarly, regarding invasiveness, the timing of phenological traits can prove crucial since it enables introduced alien species to adapt to new environmental conditions^24^. Yet, few studies evaluate phenological responses to climate between native and non-native species^25,26^. Phenological studies show us ways to tackle invasive plant growth^27–31^. However, before focusing on studying the interaction between plant species, detailed phenological studies are essential to address as they aid in restoration planning and management efforts to work effectively^27,32^.

To control the spread of IAS, traditional biological methods are prioritized^33^ over the mechanical and chemical control. Biological control has emerged as an effective tool for managing invasive species in vulnerable settings^34,35^. There are over thousand classical biological control programmes for invasive species with limited non-target impacts^36^.

**Figure 2:**
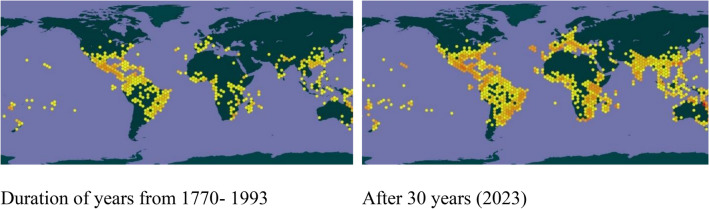
Map showing the pan-tropical presence of *Lantana* in different subcontinents. Hexagons shows the occurrence of *Lantana* indicated by the number of observations, colour coded from red to yellow. Red colour indicates a greater number of observations and yellow, fewer. The maps are adapted from Global Biodiversity Information Facility (GBIF) secretariat (https://www.gbif.org/)^37^, GBIF, 2022.

*Lantana* is a pantropical invasive weed that harms ecosystems, reduces ecosystem productivity and contributes to biodiversity loss^38^. It is considered as native to Central and South America. Figure 2 depicts its spread over the globe in past thirty years. IAS, like *Lantana,* release allelopathic chemicals (Lantadene A–D) in the soil^39^, which directly affect the growth of native plants in the vicinity or indirectly suppress growth of native plants by disrupting the soil-microbiota or altering soil resources^40^. Native plant allelopathic effects on exotic plants and interactions between conspecific and heterospecific invading plants have rarely been studied^41^. Despite the costs involved in its removal, people have been using mechanical methods to weed out *Lantana*^42^. Considering the opportunistic behavior of this weed, it reestablishes itself by finding open canopies^43^. Cutting them, spraying herbicides, or using any biocontrol agent does not work well with these species^44^. The only natural process preventing its growth is establishing canopy cover using fast-growing native trees and grasses that can claim the open land, followed by mechanical removal^45^. Numerous efforts have been made to control the spread of this invasive species through mechanical, chemical, and biological methods^46^. However, out of these methods, the biological method was found to be more economical, sustainable, and evident^47^. Studies^48,49^ have shown that fast-growing plant species can be selected to restructure a community invaded by a weed. In order to lessen *Lantana*'s invasiveness by introducing native species and mechanical eradication, researchers^35,50^ concentrated on conceptualizing an appropriate crop competitive approach. The selection of competing species with the potential vigor to resist invasive must take into account the geographic location involving edaphic components and climatic conditions in order to reduce their reemergence by creating a closed canopy^30,51^.

Interaction between plants can range from facilitative to competitive, and restoration ecology aims to promote native species flourishing in the long run to preserve biodiversity^52^. Few studies have focused on multiple plant interactions^53^ and more detailed studies are required to understand the underlying mechanisms of species coexistence. Furthermore, present studies can serve as a baseline for predicting potential phenological mismatches in species behavior in the context of climate change.

## Rationale behind the study

*Lantana* is an opportunist invasive species, which proliferates quickly to occupy open areas. The mere removal of invasive species may not suffice for ecosystem recovery due to the alterations they make to habitat conditions, rendering it inhospitable for native species. In such instances, eradication efforts should be complemented by appropriate restoration measures that ensure recovery and long term health of native ecosystems. A study^54^ proposed that after removing, it is advisable to replant the cleared areas with native species. According to field observations, selected plant species that are native to the region, grow quickly, provide a sizable quantity of biomass, and improve the soil conditions in their planted locations^55^. In addition to these advantages, these plants are used as fuel, fodder, and medicine. Understanding the biology and timing of invasive plant species phenology can help in the selection of an efficient treatment technique and its application in the field^27^ . There is a gap in relevant studies on plant interaction involving invasive species^46^. Community ecologists have focused on understanding phenology, which is crucial in assigning competitive advantages to invasive alien species. Detection, control, and mitigation of invasive species may be aided by developing phenological calendars like BBCH^56^. With this background, the objective of the present study was to monitor the growth and competition behavior of six native tree species with *Lantana* in Doon Valley of IHR followed by studying their detailed phenology. The study was conducted in three phases: (Phase 1) Identification of native species co-existing with *Lantana* in their natural habitats, (Phase 2) Study of the phenotypic traits of the co-existing species and (Phase 3) Interaction of the selected species with *Lantana* in the pot and field experiments.

## Methods and experimental design

### Experimental site

**Figure 3:**
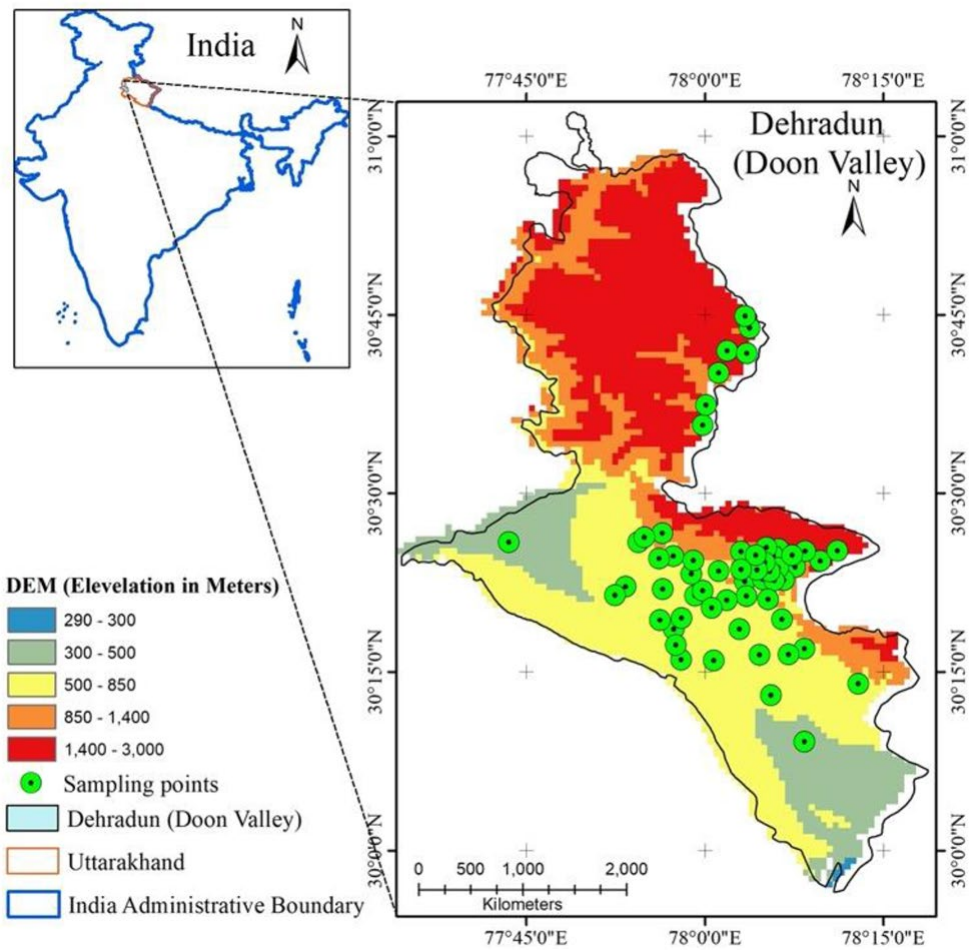
(**a**) Sample points in Doon Valley, (**b**) digital elevation map (DEM) of Doon Valley.

The survey for identification of the native species co-existing and competing with *Lantana* in their natural habitats (Phase-1) was undertaken in the Doon Valley of IHR [325–2300 above mean sea level (amsl)^57^]. The survey was done through the nested quadrat method. The species were identified with the help of revised Forest flora of Chakrata Dehradun and Saharanpur^58^. Help of taxonomist from Forest Botany Division of FRI, Dehradun was taken for the identification of unidentified species. For further confirmation, herbariums and FRI Dehradun Herbarium was consulted. However, no plant specimens were collected from the field as most of them were very common and easily identifiable plants. Through GIS-based random selection process, 60 points (GPS points) were identified within *Lantana*-infested areas in the Doon Valley (Fig. 3). We used Arc-GIS (10.1 version)^59^ software obtained from IT and GIS Division, FRI.

District map boundaries of Doon valley were obtained from Survey of India (Scale 1–50,000) used in Fig. 3. The codominant species at each of the 60 sites (Fig. 3) were monitored to study the phenological characteristics (Phase-II). DEM classes in Fig. 3 with red colour (1401–3000 amsl) did not show presence of *Lantana* due to change in the weather patterns. We surveyed along the increasing gradient and laid some sampling points to observe this pattern.

The pot experiments to study the interaction of the selected species with *Lantana* (Phase III) was carried out in the premises of Forest Research Institute (30.3315000 N, 77.9951000 E), Dehradun, India. Field trials to study the interaction of *Lantana* with the selected species was undertaken at two sites, (i) Bhopalpani (GPS 30.308300 N, 78.1196000 E) Raipur region, Mussoorie Forest Division of Doon Valley, Dehradun, India and (ii) the premises of Forest Research Institute (30.3315000 N, 77.9951000 E). Necessary permission was taken from the Divisional Forest Officer of Mussoorie Forest Division and Head, Silviculture and Forest Management (SFM) Division of Forest Research Institute (FRI) for conducting the experiments.

Doon Valley is surrounded by Shivalik range of the Himalayas. The vegetation in the region includes a mix of deciduous, lush green valleys and terraced fields. The valley has fertile alluvial soil with sandy, clayey, and rocky components. The climate of Dehradun is subtropical, humid climate and experiences four distinct seasons: Winter (December to February), Summer (March to May), Monsoon (June to September), and Post-monsoon (October–November) respectively. Mean values of climatic parameters variables like temperature, rainfall, relative humidity, specific humidity and wind speed were sourced from data access viewer: POWER by NASA (National Aeronautical Space Agency) for four years (including individual months).with an average yearly rainfall of about 168.75 mm. Doon valley receives168.75 mm annual rainfall, 70–80% of the precipitation is received during June and September.

**Figure 4:**
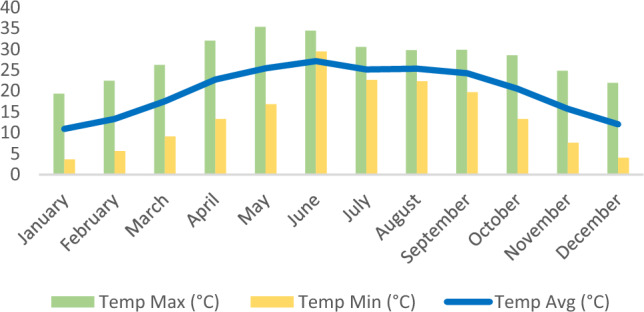
Average temperature (maximum, minimum, and average) recorded for Doon Valley in the last four years.

**Figure 5:**
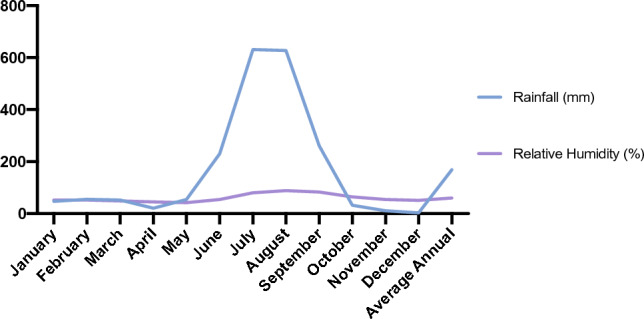
Average rainfall (mm) and relative humidity (%) recorded for Doon Valley in the last 4 years.

**Figure 6:**
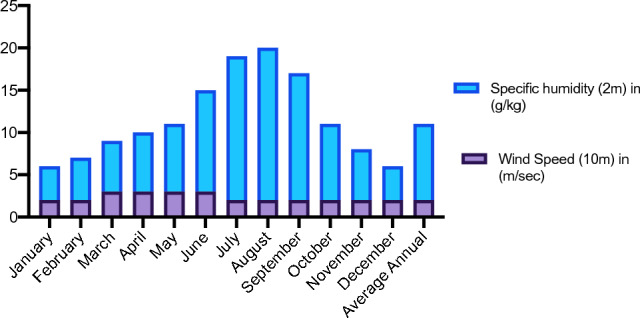
Specific humidity at (2 m) in g/kg and wind speed at 10 m in (m/s) were recorded for Doon Valley in the last 4 years.

Average annual mean for different climatic variables have been recorded for four years, i.e., wind speed (2.35 m/sec) (Fig. 6, specific humidity (9.31 g/kg) (Fig. 6), rainfall (168.75 mm) (Fig. 5), relative humidity (59.51%) (Fig. 5), temperature max. (36.27 °C), temperature min. (9.18 °C) and temperature avg. (18.11 °C) (Fig. 4). Graphs were made by using Excel (Version 16.76) and Prism 8 (Version 8.4.0) (Figs. 5 and 6).

### Sampling for identification of native species co-existing with *Lantana* in their natural habitats (Phase-I)

The vegetation of these sites was documented through clustered sampling where 10 quadrats of 10 m × 10 m were established at each site. To study the shrubs co-occurring with *Lantana* at each location, 5 m × 5 m quadrats were nested in each 10 m × 10 m quadrat. Phytosociological data was collected from each quadrat as per Ecology workbook^60^. The collected data was further analyzed for phytosociological attributes viz, density, frequency, abundance, Basal cover, and IVI following standard methods as described by Mueller Dombois and Ellenberg^61^. Data for cover-abundance of the invasive species was also collected from each site using Domin-Krajin scale^58,62^ (Table 1). The following scale value of Domin Krajin is absolute as far as the values relate to a reference area.

**Table 1:** Domin Krajin Scale for cover abundance.

Scale	Cover %	Details
10	≈100	Any number, with complete cover
9	> 75	Any number with more than ¾ but less then complete cover
8	50–75	Any number with ½–3/4 cover
7	33–50	Any number with 1/3–½ cover
6	25–33	Any number with ¼ to 1/3 cover
5	10–25	Any number with 1/10 to ¼ cover
4	5–10	Any number with 1/20–1/10 cover
3	1–5	Scattered with cover under 1/20
2	< 1	Very scattered with small cover
1		Seldom with insignificant cover
+		Solitary with insignificant cover

### Documentation of phenotypic traits of the selected co-existing species of *Lantana* in doon-valley through landmark BBCH scale (Phase-II)

BBCH scale was used to document the phenology of seven species selected on the basis of observation made in phase-I. The BBCH scale aligns with the established pattern of PGS stages, as detailed in (Table 2).

**Table 2:** BBCH codes for different PGS (0–9).

Code description	
Principal growth stage 0: sprouting/bud development	
00	Dormancy: buds closed and covered by scales
01	Beginning of bud swelling
03	End of bud swelling
07	Beginning of sprouting or bud breaking; shoot emergence
09	Buds show green tips
Principal growth stage 1: leaf development	
10	Green leaf tips 10 mm above the bud scales
	Bud elongation
11	First leaves unfolded
15	More leaves unfolded, but not yet at full size. First leaves unfolded
17	Most leaves unfolded on majority of tree
19	Leaf expansion complete
Principal growth stage 3: stem elongation	
30	Beginning of stem elongation
31	Stem about 10% of final length
33	Stem about 30% of final length
35	Stem about 50% of final length
35a, 35b, 35c……	Stages continue
39	Stem about 90% of final length; cessation of stem growth
Principal growth stage 5: inflorescence emergence	
51	Inflorescence or flower buds visible
55	First individual flowers visible but still closed
59	First flower petals visible (in forms with petals
Principal growth stage 6 : flowering (main shoot)	
60	First flowers open
61	Beginning of flowering, 10% flowers open
62	20% of flowers open
63	30% of flowers open
64	40% of flowers open
65	50% of flowers open, full flowering: first petals may be fallen
67	Flowering finishing; majority of petals fallen or dry
69	End of flowering: fruit set visible
Principal growth stage 7 : fruit development	
72	Fruit 20% of final size
75	Fruit 50% of final size
78	Fruit 80% of final size
79	Fruit final size
Principal growth stage 8 : fruit ripening	
89	Fruit fully ripe
Principal growth stage 9 : senescence, beginning of dormancy	
91	Shoot growth completed; foliage still green and terminal buds developed
92	Beginning of leaf discoloration
93	Beginning of leaf fall
95	50% of leaves fallen
97	End of leaf fall

While BBCH scales have previously been documented for *Aegle marmelos* (AM)^63^, *Lantana* camara (LC)^64^, and *Broussonetia papyrifera* (BP)^65^, it is important to note that such a scale was hitherto unavailable for *Pterospermum acerifolium* (PA)*, Urtica dioica* (UD), *Pongamia pinnata* (PP)*, Justicia adathoda* (JA), and *Bauhinia variegata* (BV). To precisely monitor vegetative and reproductive phases during the growing period, periodic visits were made every two weeks. However, during the critical growth phase (phase change to reproductive growth), observation frequency was increased to once every week, as per the requirement daily observations were also made to report minute changes in phenology. The details of the BBCH scale is presented in Table 2. A phenophase was reported only if > 75% of the individuals in the area were showing the required character.

### Study on the interactions of the selected species with *Lantana* in the pot experiments (Phase-IIIa)

Seven co-dominant native species i.e., UD*,* JA*,* BP*,* PP*,* BV*,* PA *and* AM were chosen to proceed with pot experiments followed by Phase-I and Phase-II observations. This is to further clarify that none of these species is enlisted in IUCN list of threatened species or CITES list. We performed these experiments to explore the competitive dynamics between *Lantana* and seven native plant species. To assess these interactions, 15 distinct treatments was designed. Among these treatments, seven investigated interspecific competition, and the remaining eight assessed intraspecific competition.

In these experiments, young plants from each species were paired with *Lantana* in a 1:1 ratio to study interspecific competition (native species with *Lantana* were planted in the pot); similarly, two individuals of the same species were planted in the pot to study intraspecific competition. These plants experienced same water and nutrient availability conditions during the experimental tenure. The growth and resource allocation were evaluated in the form of wet biomass, which effectively indicated their competitive effects on each other under same edaphic conditions. The study commenced with seed sowing in June 2019, and all plants grew under similar conditions within the nursery at the New Forest Campus, SFM Division, Forest Research Institute, Dehradun. To ensure adequate moisture levels, the pots were consistently watered during the summer months and three times per week during other seasons.

Furthermore, to prevent competition among the pots, they were randomly arranged with a minimum spacing of 40 cm between each of them. Although, some plants in the experiments could not survive due to disturbance by wild animals, wind, or breaking of pot. The plants were harvested from the individual pots on September 2022. Each individual from the pot was cut into smaller pieces, weighed separately using electronic weighing balance to record the wet biomass of each individual. The data thus collected was used to determine Relative Interaction Index of each species.

### Relative interaction index (RII)

RII was used as a comparative index to measure the competition between selected natives and *Lantana*. It is suitable for parametric meta-analyses due to its strong statistical properties This index measures the facilitative or competitive interaction between plants^66^. Negative values indicate that the interaction between plants is competitive and positive values indicate that interaction is facilitative. RII = 0 suggests no significant change of mixed monoculture on the plant's growth. The formula is also represented as Bw = Bo + ΔBF – ΔBC, where Bw is biomass observed by the target plant grown with other plants (treatment), and Bo is biomass potentially achieved without species interaction (control).

$$\begin{aligned} {\text{RII }} & = \, \left( {\Delta {\text{BF}} - \, \Delta {\text{BC}}} \right))/\left( {\left( {\Delta {\text{BF}} + {\text{Bo}}} \right) \, + \, \left( { - \Delta {\text{BC}} + {\text{Bo}}} \right)} \right) \\ & = \, \Delta {\text{BFC}}/\left( {\Delta {\text{BFC}} + {\text{ 2Bo}}} \right) \\ & = \, \left( {{\text{Bw}} - {\text{Bo}}} \right)/\left( {{\text{Bw}} + {\text{Bo}}} \right),{\text{ RII }} = \, \left[ { - {1},{ 1}} \right] \\ \end{aligned}$$

ΔBF is denoted as an increase of biomass produced by facilitation (from 0 to + ∞), and ΔBC is a decrease in biomass due to competition. ΔBFC = ΔBF – ΔBC, i.e., observed biomass change = absolute effect of the interaction. Therefore, Bw − Bo = ΔBFC. Facilitation and competition would not be compared using these equations. RII, is proposed as: (treatment − control)/(treatment + control). These experiments were carried out consecutively for four years, and data were recorded for the phenological growth stages.

### Study on the interactions of the selected species with *Lantana* in the field experiments (Phase-IIIb)

The same set of selected species were planted in field at two different geographical locations in Doon Valley to observe the performance of species under varying field conditions. Nursery-grown seedlings of identified plant species were used for plantation after the mechanical removal of *Lantana* from the field sites. Outplanting was chosen rather than seeding because the survival rate of outplanting is higher. Seeds were collected from the plants growing at the experimental site in FRI. The seedlings for the experiments were raised in nursery of SFM Division of FRI. Necessary permissions were taken from the Head, SFM, Division for conduction the experiments. Seeds were sourced from the FRI campus itself and none of the plants falls in rare/endangered/threatened categories. Proper permissions from the SFM Division were taken for seed collection. The authors comply with the IUCN Policy Statement on Research Involving Species at Risk of Extinction and the Convention on the Trade in Endangered Species of Wild Fauna and Flora. The experimental field infested with *Lantana* was chosen for the experiment, the *Lantana* was cleared through mechanical removal. Wet Biomass of *Lantana* and other native species was recorded for each plots. Following the three-tier vegetation system, grasses were introduced to coverup the open area after removal of *Lantana*^67^. Seeds of four grass species viz. *Pennisetum pedicellatum, Panicum antidotale, Sachharum spontaneum,* and *Sorghum halpense* were mixed in equal proportion (500 g seeds of each species) and seeds ball were prepared. These native grasses were sourced from the adjacent field areas and were used to reclaim the study sites after mechanical removal of *Lantana*. For preparing seed balls, soil, clay and farm yard manure were mixed in the ratio of 2:1:1 to form 10 kg of this mixture. Seeds were later added to this mixture, and mixed throughly. Seed balls were prepared and dried in shade and spread in the field in equal distance (30 cm) in July,2019. The research hypothesis was fast-growing grass would cover the field and prevent the regrowth of *Lantana.* Two field experiments were conducted using a Randomized Block Design, consisting of eight treatments in total. These treatments included seven native species individually, each replicated five times, and a control plot where *Lantana* was not removed. Each treatment was represented by block plantation of 16 plants of the selected species planted at 1.5 m × 1.5 m distance. The plantation was undertaken in the first week of August, 2019. *Lantana* was not planted as root suckers and seeds of the species were already present in the field. After three years of growth the biomass from each plot was harvested and wet biomass was measured for the grass species, native species and *Lantana*.

## Results and discussion

### Native species co-existing with *Lantana* in natural habitats (Phase-I)

**Figure 7:**
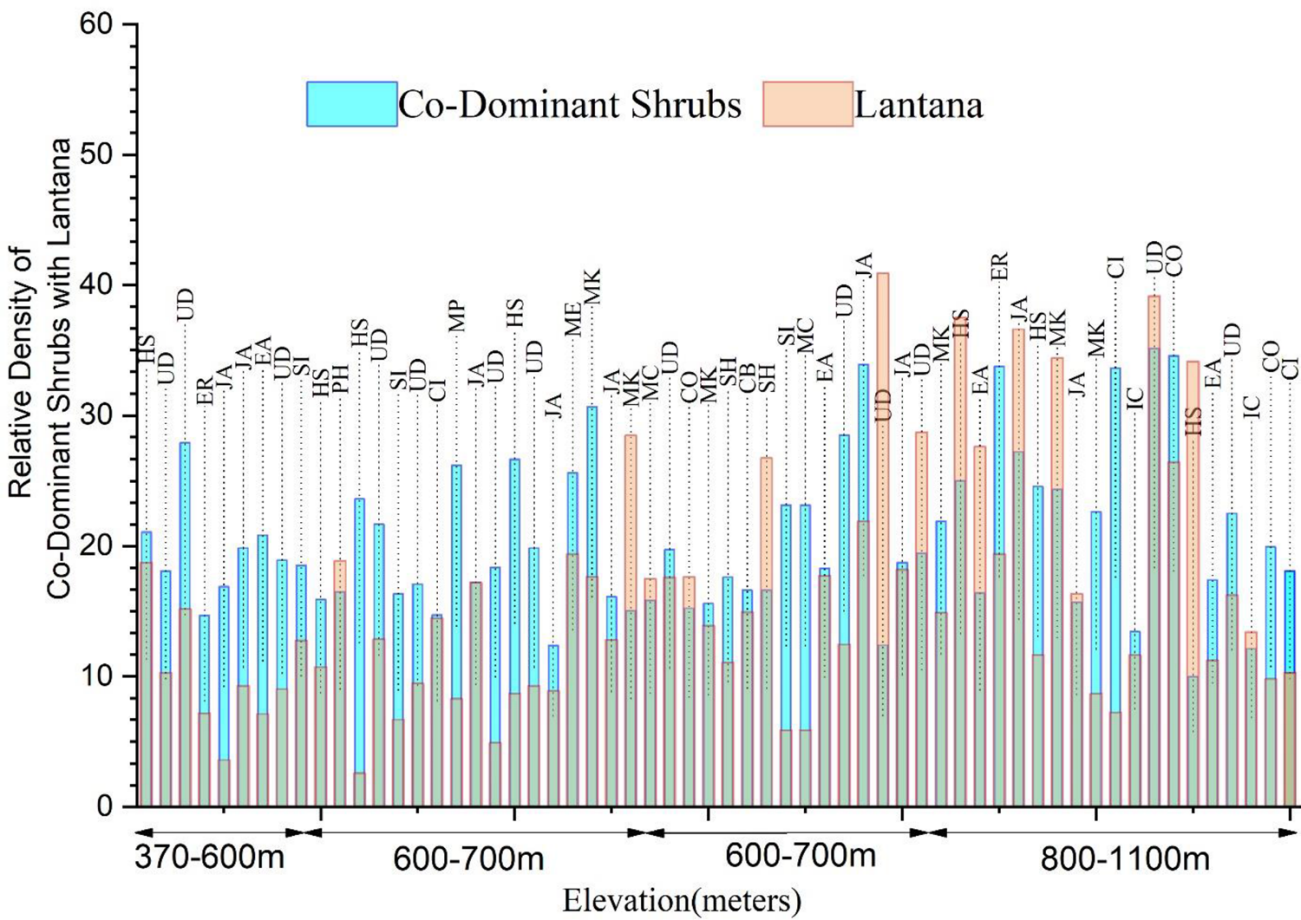
Graph represents relative density of co dominant shrubs with *Lantana* with respect to elevation (in metres).

*Lantana* shares the habitat with an array of IAS and native species, the altitude wise relative density of the *Lantana* and co-dominant species is presented in Fig. 7. Major native shrub species found with *Lantana* are *Justicia adathoda* (JA)*, Urtica dioica* (UD)*, Euphorbia royleana* (ER)*, Solanum indicum* (SI)*, Clerodendrum infortunatum* (CI)*, Mallotus philippensis* (MP)*, Milletia extansa* (ME)*, Murraya koenigii* (MK)*, Malvastrum coromandelianum* (MC)*, and Colebrookea oppositifolia* (CO)*,* while the co-dominant exotics comprised of *Solanum hispidum* (SH)*, Eupatorium adenophorum* (EA)*, Parthenium hysterophorus* (PH)*, Hyptis suaveolens* (HS) and *Ipomea carnea* (IC)*.* It's worth noting that *Lantana* exhibits higher dominance than co-dominant species in only 15 sites. At 33 sites native species were dominant (IVI higher than *Lantana*); at 12 sites, different IAS were dominant species.

**Figure 8:**
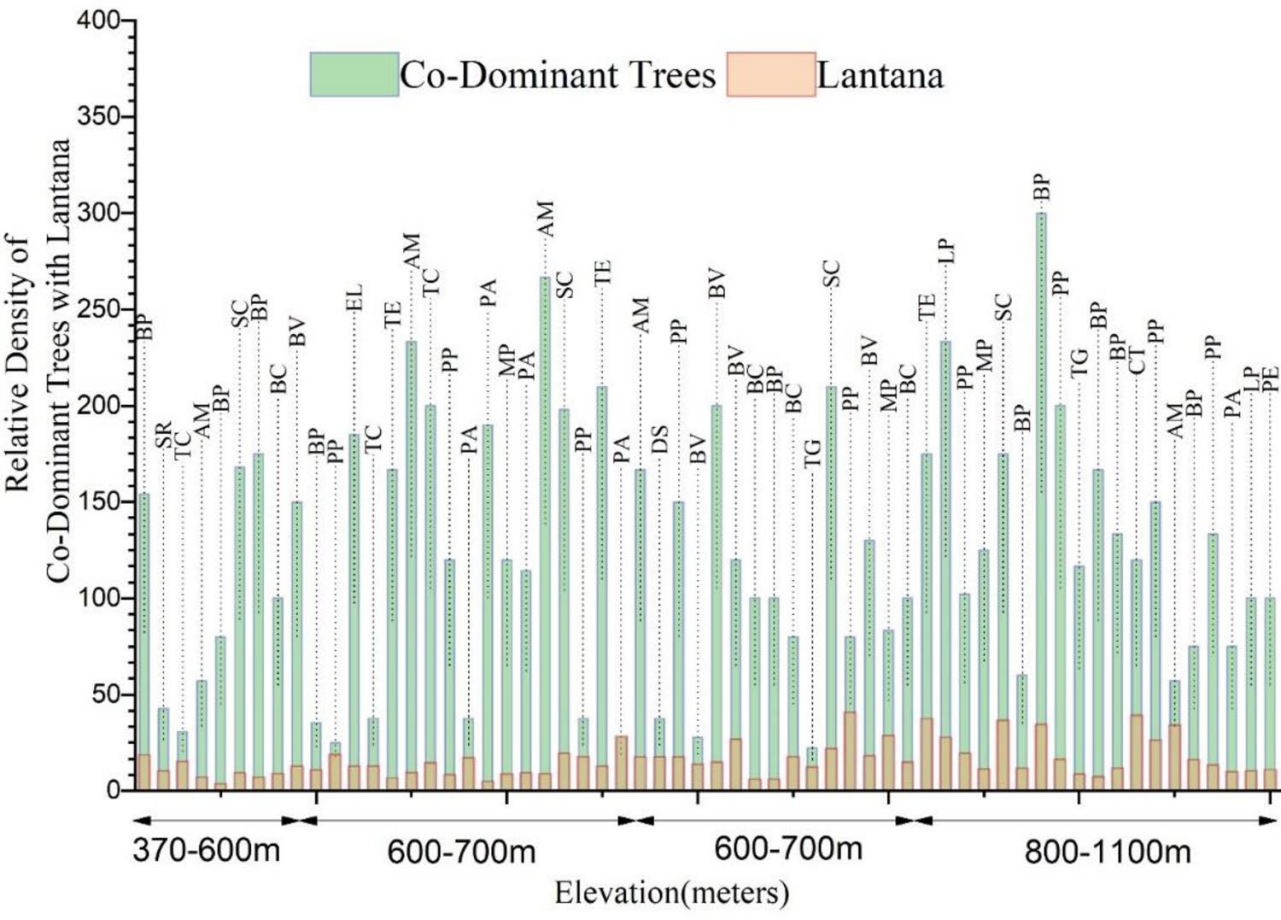
Graph represents relative density of co dominant trees with *Lantana* with respect to elevation (in metres).

Seven locations out of 60 sites were reported with co-dominant exotic tree species with *Lantana* (Fig. 8)*.* Native tree species such as *Broussonetia papyrifera* (BP), *Shorea robusta* (SR), *Aegle marmelos* (AM), *Bauhinia variegata* (BV), *Pongamia pinnata* (PP), *Ehretia laevis* (EL), *Terminalia elliptica* (TE), *Pterospermum acerifolium* (PA), *Mallotus phillipensis* (MP), *Syzium cuminii* (SC), *Dalbergia sissoo* (DS), *Tectona grandis* (TG), *Lagerstroemia parviflora* (LP), *Caseaseria tomentosa* (CT), *Phyllanthus emblica* (PE), *Bombax cieba* (BC) and only one exotic tree species *Toona ciliata* (TN) was recorded from the field observations.

**Figure 9:**
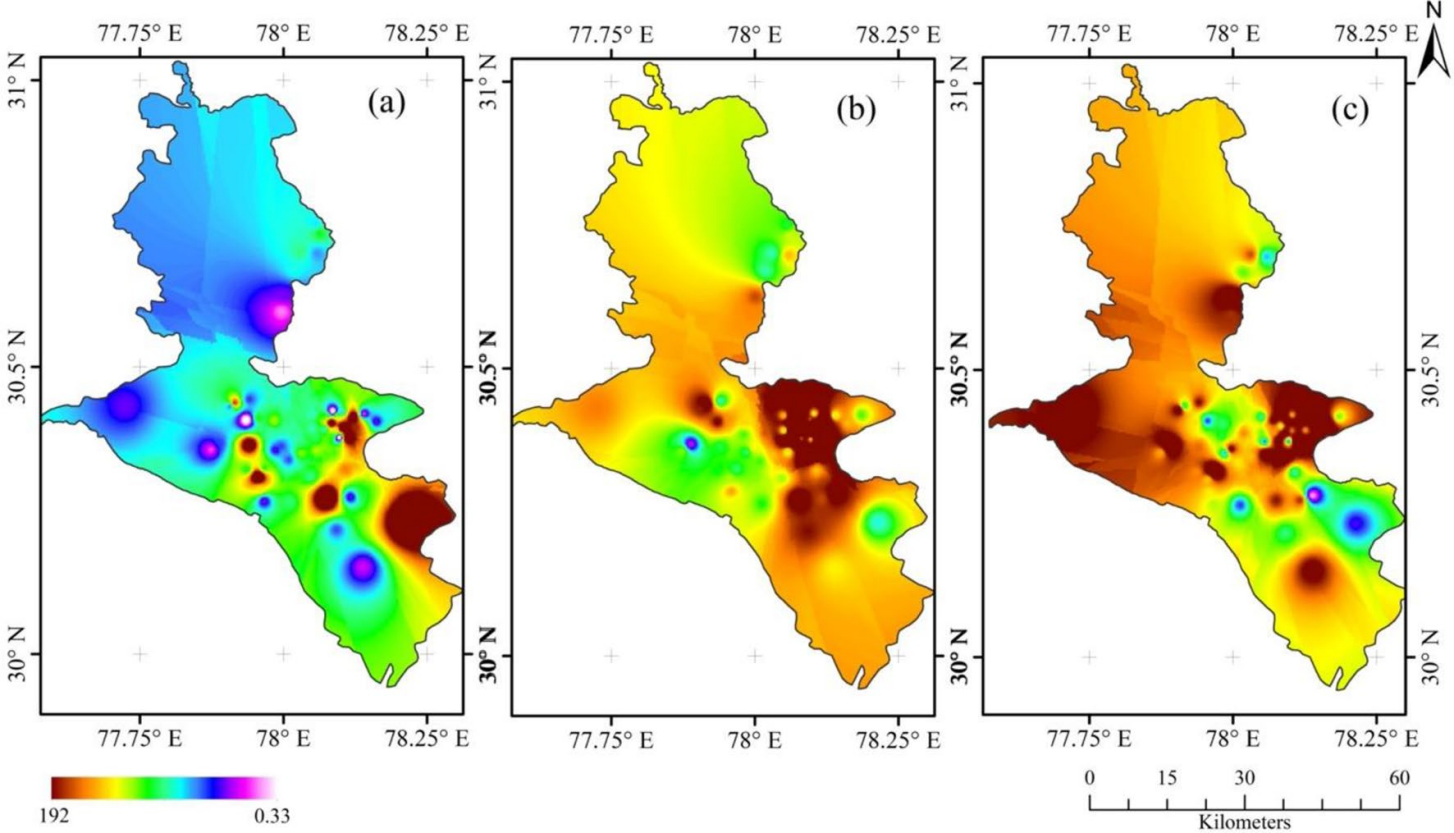
Map showing colour-coded raster layers depicts the Importance Value Index (IVI) for (**a**) *Lantana*, (**b**) co-dominant shrubs, and (**c**) dominant trees in the Dehradun district at various survey locations, with values ranging from lowest 0.33 to highest 192.

The analysis of phytosociological data collected from the survey shows that *Lantana*'s dominance varies at different locations. The IVI values were calculated after Quadrat surveys in different locations of Doon Valley. The IVI for *Lantana* in the Doon-valley ranged from 0 to 192, the dominance of *Lantana* decreases with the increase in altitude, as depicted in the Fig. 9. Maps in Fig. 9a–c were generated to depict the IVI of *Lantana*, co-dominant shrubs and dominant trees in *Lantana* infested areas in Doon Valley respectively. These GIS maps collectively provide a comparative assessment of the extent of *Lantana* invasion in the region. Brown layer indicates areas where *Lantana* has high dominance value (IVI), mostly found in the lower Doon valley's lower altitude. The spread of *Lantana* in Doon Valley can be seen clearly with the prevalence of green colour that signifies the mid-range of IVI value. The lower half of the Doon Valley map has a lower elevation and is heavily infested by *Lantana*, according to the survey, except for some areas that range from light blue to pink. Through the further inspection of Fig. 9, it can be inferred that *Lantana* and other shrubs thrive in the lower half of Doon Valley (250–850 m), particularly in the central region (Fig. 9b) marked by green and yellow. This observation supports the theory that *Lantana* avoids shaded areas under tree canopies, preferring to expand in well-lit, open environments^68^.

### Phenotypic traits of co-existing species in the Doon Valley (Phase-II)

**Figure 10:**
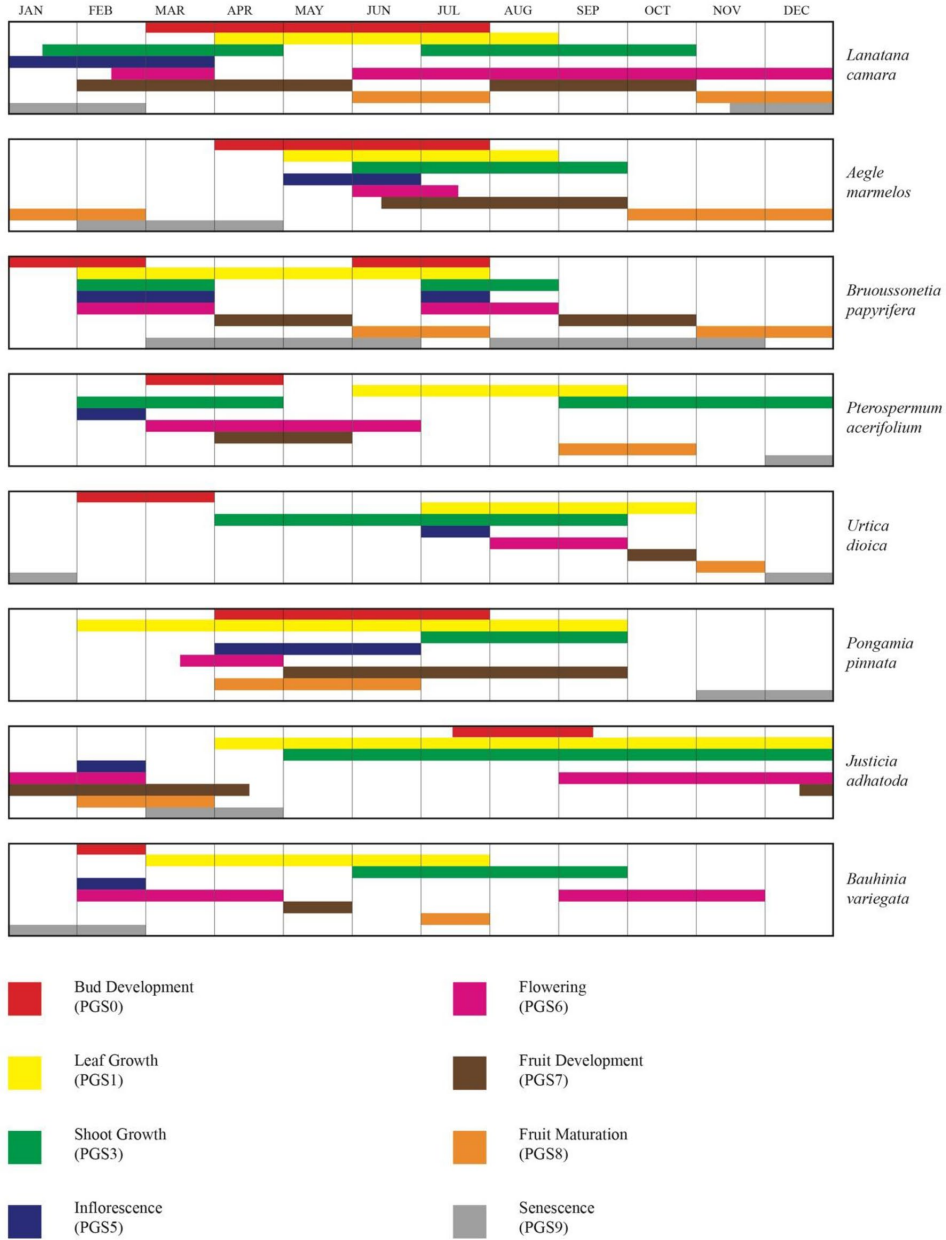
This figure represents a seven-species phenology comparison following the BBCH scale. Different PGS stages (0–9) for other plant species have been illustrated by different colour codes in the respective month of their occurrence. BBCH scale for PA, UD, PP, AV, and BV are introduced here for the first time.

The scale uses bi-numeric codes to indicate important Principal Growth Stages (PGSs, 0–9) and related secondary growth stages (SGSs, 0–9). Based on the observation during phase-I five tree species (AM, BP, PA, PP, BV) and two shrub (JA, UD) species were selected for further phenology study. Temperature and rainfall plays an important role in governing the phenology of plant species^69^, therefore it is important to include such factors while studying phenology^70^. Wind speed and specific humidity^71^ are also studied^72^ as they are intricately linked to climate patterns and phenological shifts^73^. Variation in a plant's phenology is caused by ongoing environmental changes linked intricately to all these factors like specific humidity (observed high while June–September, Fig. 5) and wind speed (observed high during March–June, Fig. 5). The associates of the Lantana showed varying phenology in the study area. The phenophase of flowering in *Lantana* has two peaks (mid February–March and June–December), the fruiting phase is prominent from June to December. Flowering and fruiting patterns in BP, UD and BV coincide with *Lantana*’s timing (Fig. 10). Whereas, difference in timing of flowering and fruiting is observed in PP (flowering in April, fruiting in June–July) and PA (flowering and fruiting in April–May) (Fig. 10). Table 3. summarizes the life history traits of the studied species. Few studies focus on the phenological interactions between natives and invasives in the same environmental conditions^74^. Phenological duration is a crucial factor in decoding the timing of individual species and gives us insight on the timing for introducing native species in the field^24^. Changes in the phenology of these native species coupled with climatic variables lead ecologists and researchers to design and implement restoration plans for invasive species like *Lantana*^75,76^.

**Table 3:** Life history traits for all the species observed during the experiments.

Feature	*Lantana camara*	*Justicia adhatoda*	*Urtica dioica*	*Bauhinia variegata*
Life forms	Scrambling aromatic shrub	Small evergreen, sub-herbaceous shrub	Perennial herb	Medium sized tree
Habit	Erect in the open and scrambling in scrubland, perennial	The plant grows in plains & lower Himalayan ranges	Extensive sympodial system of Rhizomes and stolon, rooting at the nodes	Distributed throughout India and avoids closed canopies
Plant height	Usually up to 2 m but can grow to the height of 5 m	Upto 2.5. Can grow upto 3 m in height	Aerial Shoots (up to 1.5–2 m). Can attain 3 m or more height sometimes	It can grow up to 20–40 feet tall and attain canopy of 25–30 feet
Stem	Woody squarish in cross-section and hairy when young. Becomes cylindrical upto 150 mm . Gets thick with age	Long Opposite yellowish , ascending branches	Herbaceous stems with four angles are typically erect, unbranched, and up to 6 feet tall. They are covered in stinging hairs	The inner bark is pinkish and fibrous, whereas the outside bark is scaly, smooth to slightly fissured, and brownish-grey
Leaf shape	Ovate entire with serrate margin	Opposite, minutely pubescent	Heart-shaped opposing leaves with serrated margins and many stinging hairs resemble mint. Leaf base is cordate to rounded	Cow foot Shaped leaves
Leaf arrangement	Opposite pairs at nodes all along the stems	Broadly lanceolate	Opposite	Bilobed and alternate
Inflorescence and color of flowers	Axillary heads; flowers ranging in color from cream to yellow to orange, pink, purple and red	Dense, short pedunculate, spike, bracteates with long bracts	Axillary, spike like :four per node	Raceme, light pink to pale Purple
Flowering period	September to May, flowers throughout the year subject to the availability of water	Early Sep–late Feb	August to September	September to November
Flowers per plant	20–40 flowers per head	5–35 flowers per spike	20–60 flowers per head	–
Fruit	Fleshy drupe, 3–6 mm in diameter and containing, 1–2 seeds (12.5 mm long). Fruits mature rapidly and change color from dark green to black	Fruits are pubscent and club shaped capsules, longitudinally channelled	Fruits single seeded, Achenes small	Fruit is a dehiscent pod that is strap-shaped, 15 to 30 cm long, and contains a variety of flat seeds
Variability in seed production	12,000 seed per plant	After 2 years, flowering and fruiting become regular	500–5000 seeds per shoot	After 2 years starts producing flowers
Gestation period	January, May and November	In between March to December	September–November	April–August

Feature	*Broussonetia papyrifera*	*Pongamia pinnata*	*Pterospermum acerifolium*	*Aegle marmelos*
Life forms	A deciduous tree. It has a wide, spreading crown	Fast-growing evergreen tree. Branchlets have light stipule scars and no hair	The evergreen tree has a crooked crown and dense, sharply ascending branches	Deciduous tree
Habit	Large tree	Large tree	Large tree	Tree
Plant height	Can reach a height of 15 m. , and under the right circumstances, it may even reach higher	It can reach 25 m (65 feet) or more	It can reach up to 30 m	6–10 m
Stem	Strong, spreading, brittle, covered in stipular scars, and with pubescent shoots when young. Contains a milky sap	Thin grey to greyish brown and yellow on the inside	The tree's bark is relatively soft and grey in colour. Small twigs have feathery tips and are often rusty-brown	The stem bark is shallowly wrinkled, bluish-grey, and 4–8 mm thick. Smooth bark
Leaf shape	Alternate, unusual pinnately complex, hairless, 2 to 4 inches, evergreen	Hemispherical dark green leaves	Leaf edges are commonly dentate (toothed) or irregularly lobed	Alternate leaflets, single, or compound leaves
Leaf arrangement	Oval to lobed to mitten-shaped, dull green, simple, alternate (occasionally opposite or whorled), serrate margins up to 8 inches long, rough on top and hairy below leaves	Imparipinnate, 15-25 cm; leaflets opposite, elliptic	The leaves upper side has a glabrescent texture and a dark green tint. Many of the leaves tend to droop downward, making the tree appear to be wilting	Deciduous, alternating, and either single or compound bearing, leaves. In compound leaves, the leaflets have 2 to 5 frivolously toothed, pointy, oval-ovate or ovate shapes
Inflorescence and colour of flowers	The female flowers in the pistillate inflorescence are greenish, and their long styles trail behind them in a spherical head that is up to 2 cm broad Colour of the flower is Yellowish- White	Axillary racemes Colour of flowers is white to pink and purplish	Due to their outstanding fragrance and nocturnal behavior, the flowers draw moths for pollination. Flowers that are successfully pollinated generate a fruit in the shape of a tough capsule White flowers	Axillary and terminal inflorescences that are racemose or corymbose Colour of flower is greenish white
Flowering period	Feb–March and July–August	March, April	May–August	June to August starting
Fruit	Achenes that are 1–2 cm long and wide and dangle on long fleshy stalks make up the compound, shiny-reddish fruit	Numerous, elliptical, rigid, and woody indehiscent pods are the fruits	The fruit is quite rough and often has brown hairs on it. Fruits can mature for a longer time. When the capsule splits apart, many "winged seeds" are released	Fruits are red to orange, globose
Variability in seed production	540,000 seeds per kilogram	0–30 seeds/kg	12,000 seeds per kilogram	300–400 fruits per year and 10–50 seeds from each fruit
Gestation period	July–August	April–May	Sep–Dec	Oct–March

### Phylogenetic analysis

**Figure 11:**
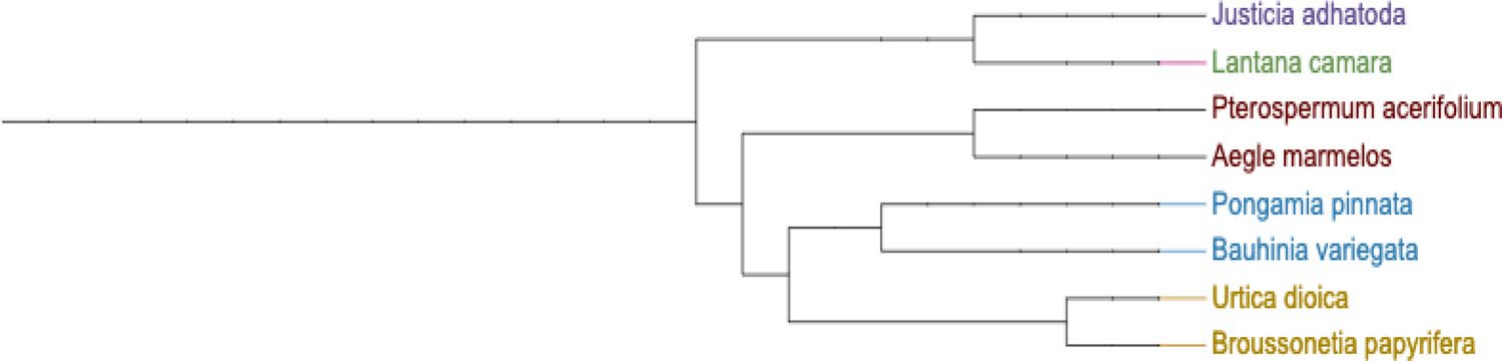
A phylogenetic tree generator, based on NCBI taxon visualized in IToL (Interactive Tree of Life). Created in Phylot V2.

Studies indicate that the species exhibiting phenotypic similarity are evolutionary related^77,78^. Phylogenetic analysis of selected species was done to trace their evolutionary relationships using Phylot software^79^ (Fig. 11). It is observed that JA has close relatedness and share the same evolutionary relationships. It is also found in nature to co-exist with *Lantana*. Similarly, BP, BV and PP are phylogenetically distant but are also found to co-exist with *Lantana*. The species sharing the same niche space over time adapt to co-exist or compete with each other. Conversely, distantly related species may have distinct resource requirements, which may facilitate coexistence^80^. Beyond resource competition, phylogenetically related plants can showcase unique adaptations that allow them to exploit distinct ecological niches, fostering coexistence through niche differentiation. Similarly, the phylogenetically distant species might deploy indirect competition and may prove to be a better competitor. The extent to which phylogenetic diversity influences or results from species assembly processes is still a matter of debate^81^.

### Interactions of selected species with *Lantana* (Phase-IIIa)

**Figure 12:**
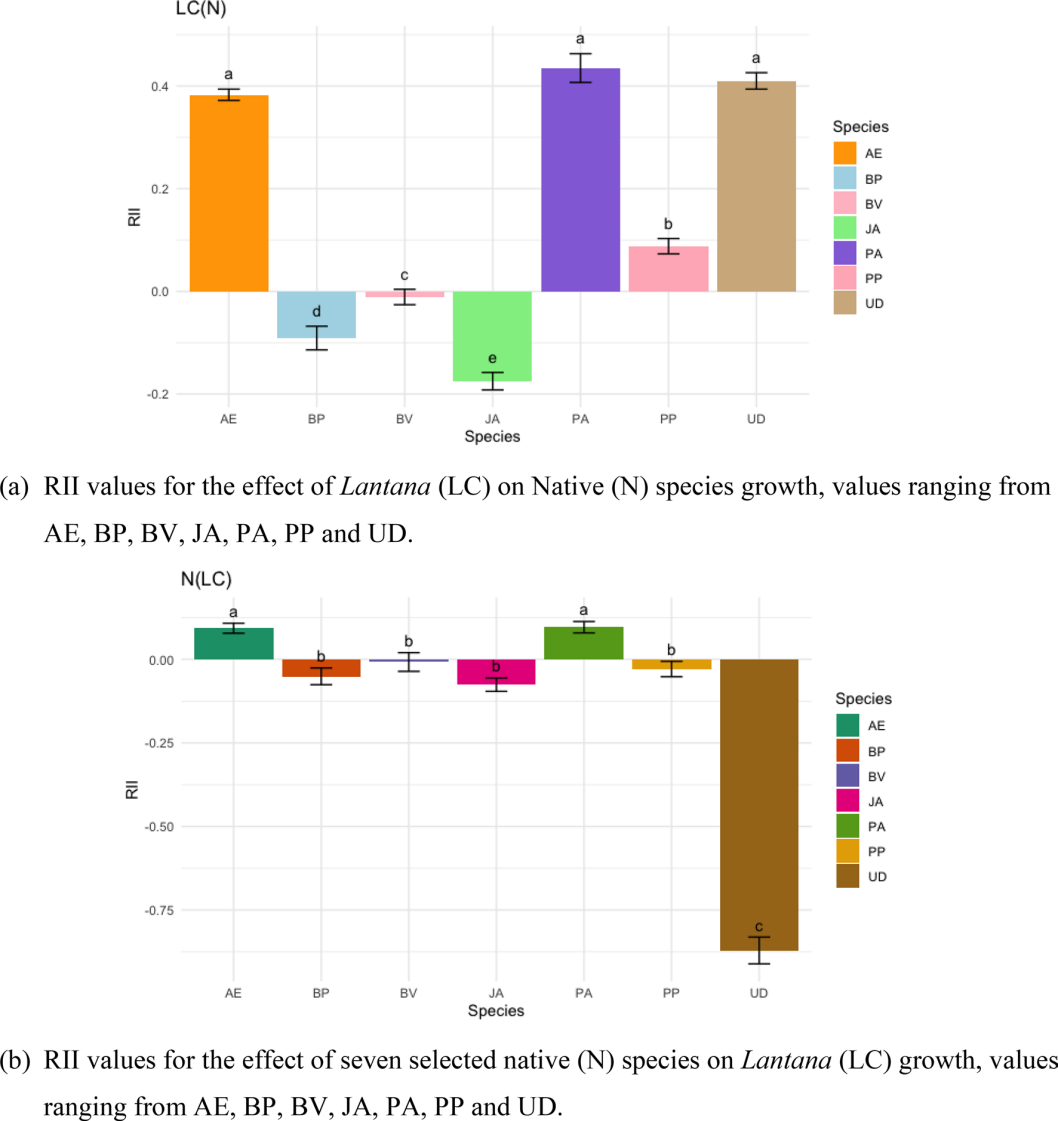
(**a**) RII values for LC(N) and (**b**) N(LC) for seven species competing with *Lantana* and vice versa.

RII value represents the interaction between the selected native species with the *Lantana*. RII graph depicts JA, BP, PP, and BV exhibited accelerated growth in the pots and have a negative impact on the growth of *Lantana*. Figure 12a indicate the competitive interaction of *Lantana* with the native species (values ranging from −0.175 to 0.435). The positive value for the interaction of *Lantana* with UD shows that UD is not impacted by presence of *Lantana*, similar observation were noted for PA and AE. Both the species showed relatively slow growth in the pots and have neither positive nor negative effect of *Lantana* on them. *Lantana* exhibited competitive interaction with PP, JA, BP and BV to varying degree.

Similarly, RII values ranged between −0.871 and 0.096 in Fig. 12b, indicating the competitive interaction of native species with *Lantana*. The high negative RII value of UD indicates the higher competitive ability of UD on *Lantana*. UD inhibited *Lantana*'s growth but did not allow it to establish in some pots. This may be due to the allelopathic effect reported from the species^82,83^. *Lantana* is reported to exhibit allelopathy^84^, but in this case, the UD appears to have a stronger allelopathic effect on *Lantana*.

### Interactions of selected species with *Lantana* in natural field conditions (Phase-IIIb)

**Figure 13:**
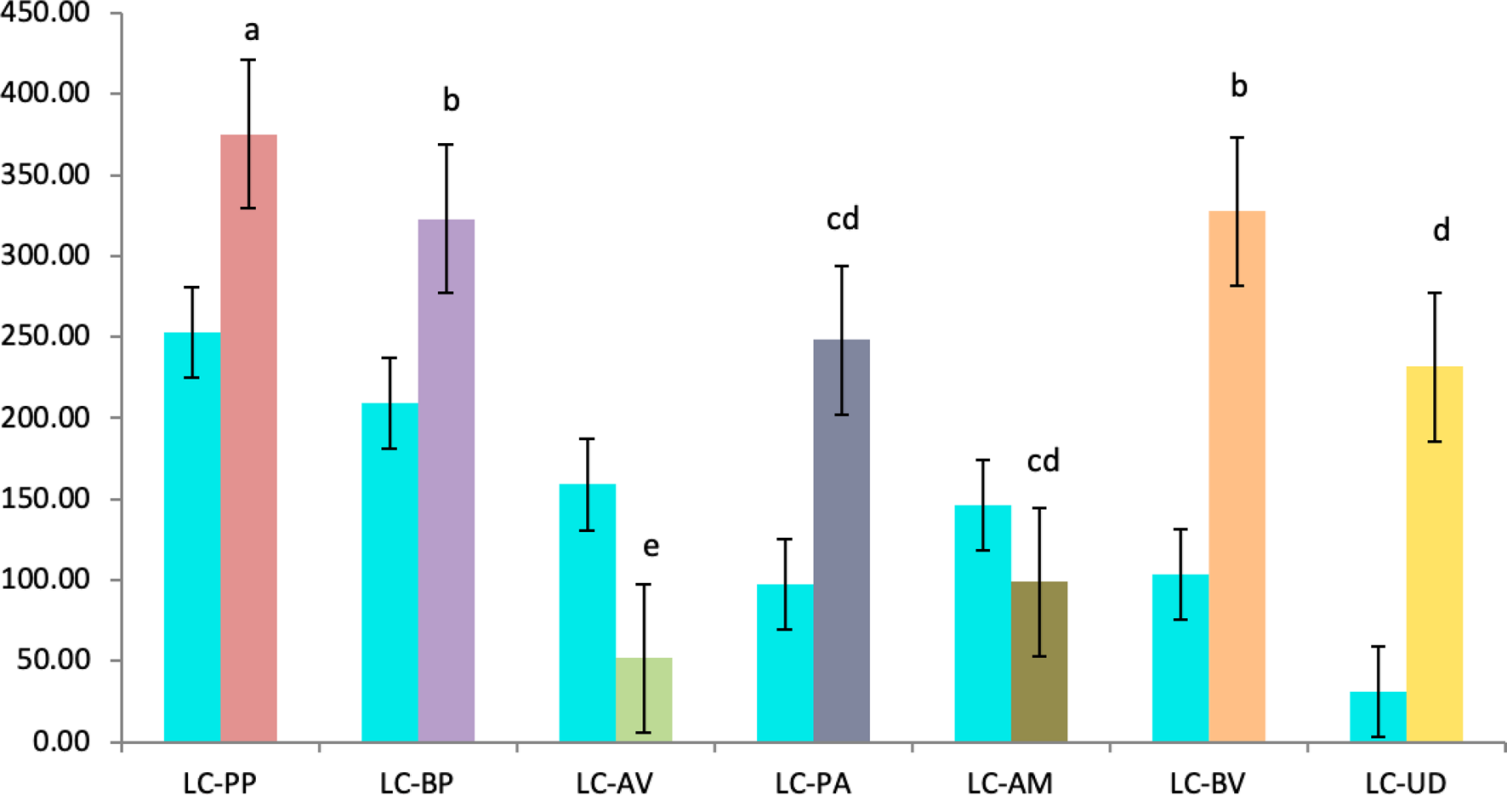
Mean values for each species (in grams) with respect to *Lantana* on x-axis. Blue represents *Lantana* growth with respect to other plants in different colors.

Among the selected species, BP, UD, PP, PA and BV demonstrated superior growth compared to *Lantana* in field conditions, as indicated by the mean wet biomass values (Fig. 13). This may also be due to the dominance of grass in the field. Due to the early growth and establishment, the grass grew taller before the seedlings of *Lantana* emerged. Thus, the seedlings of *Lantana* had to compete with grass to occupy the space leading to the development of slender, unbranched individuals of *Lantana*. At the same, the native species had a competitive advantage over *Lantana* and exhibited accelerated growth in the presence of grass.

**Figure 14:**
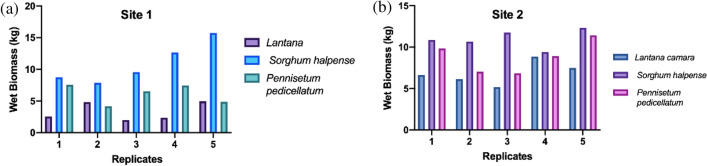
(**a**) Mean wet biomass of *Lantana* with *Sorghum halpense* and *Pennisetum pedicellatum* observed from field site 1 and (**b**) site 2 in five replicates.

The field experiment results resonated the observations from the pot experiments, except for PA, which exhibited higher mean values (Fig. 13). It is known that field conditions present distinct challenges compared to controlled pot conditions due to additional environmental variables influencing plant growth. This difference may be attributed to the improved performance of root system architecture in the field setting as opposed to pots. While UD thrives in shaded environments, *Lantana*, on the other hand, prospers in open canopies. Although UD proves to be a potential competitor (as seen in Figs. 7 and 12), its preference for shady conditions limits its feasibility for experiments in open natural settings. Nevertheless, it provides insight into leveraging allelochemicals found in UD. Extracting these chemicals can pave the way for designing a biological control approach, which could involve spraying or injecting them onto *Lantana.* Of the four types of grass used in our experiments, two species of grass, i.e., *Pennisetum pedicellatum* (PD) and *Sorghum halpense* (SH)*,* were found in abundance and thus exhibits success in controlling *Lantana*’s growth in the initial stages. Average wet biomass values indicate that PD and SS outperformed the *Lantana* (Fig. 14).

It was also observed that these grasses showed growth beyond the quadrats in which the seed balls were introduced. This may be because heavy rainfall in the monsoon season uniformly distributed their seeds in most of the field area. Apart from this SH behaved as an opportunist species and grabbed the opportunity to expand in the field, limiting growth of *Lantana*. It must be due to the seeds introduced by extraneous environmental factors and its presence in the nearby areas of the field sites. SH finds its use as a fodder species and has the potential to compete with *Lantana*. Surprisingly, PD again took hold of the other grasses in January and February and flourished well in the field. While SS being a perennial grass formed tufts and stayed. Results reveal that SH has higher values of fresh weight when compared to PD, and *Lantana* was suppressed by their presence. *Sachharum spontaneum* also performed well in the field but we did not include it in our analysis as it was not present in all the studied quadrats.

## Conclusion

Understanding IAS ecology and timely control measures are crucial for restoring degraded areas. The plant species selection and timing, based on phenological assessments, results in successful outcomes. Results reveal that different native plants exhibit differential competitive ability. UD a shade-loving allelopathic native shrub, that exhibited high potential to outcompete *Lantana*. Since *Lantana* is a light demander, it does not occupy the same niche as UD, but additional research into the species allelopathic characteristics could help manage *Lantana*. Combining mechanical removal of invasive species, reclamation of the open area with native grass, and subsequent introduction of selected shrubs and trees for increased canopy cover has proved to be an effective management strategy in the field. This study corroborates to (Sustainable Development Goal) SDG 15: Life on Land. Target 15.8, states that measures must be implemented together to prevent the introduction and reduce the impact of invasive alien species. The new insights from the present study can help the policymakers, farmers, stakeholders, researchers and conservationists in adopting the suggested strategies for invasive species management in the IHR.

## Data Availability

Datasets will be made available on request by the author Abhishek Kumar (E-mail: abhishek259kumar@gmail.com).
